# DEFINING METABOLIC BALANCE BETWEEN SUGAR AND FAT METABOLISM, AND THEIR ROLES IN DEVELOPMENT AND FERTILITY”

**DOI:** 10.1093/geroni/igad104.1135

**Published:** 2023-12-21

**Authors:** Mike Henne

**Affiliations:** University of Texas Southwestern Medical Center , Dallas, Texas, United States

## Abstract

Animals store excess nutrients in two major forms: triglycerides (TGs) held within lipid droplets (LDs) and carbohydrates in the form of glycogen. These nutrient reserves can serve specialized roles in animal development and homeostasis, but how they uniquely contribute to animal growth, bioenergetics, aging, and fertility is poorly understood. Furthermore, it is unclear how loss of one nutrient storage pool influences the other, and whether metabolic remodeling can occur to bypass loss of fat or carbohydrates. Here, we utilize Drosophila as a genetically tractable model system to dissect the tissue-specific contributions of fat and glycogen storage in animal development and fertility. We define metabolic remodeling in carbohydrate metabolism that occurs in Drosophila models nearly devoid of fat. Intriguingly, we find that loss of specific LD subpopulations in the Drosophila fat body lead to metabolic remodeling of carbohydrate metabolism, as well as alterations in female fertility and development. Our work indicates functional crosstalk and active sensing between fat and carbohydrate pools, and indicates that animals can re-wire nutrient storage pathways to facilitate development.

